# Access to Genetic Counselors in the Southern United States

**DOI:** 10.3390/jpm9030033

**Published:** 2019-07-01

**Authors:** Catalina Villegas, Susanne B. Haga

**Affiliations:** Center for Applied Genomics & Precision Medicine, Duke University School of Medicine, 304 Research Drive, Durham, NC 27708, USA

**Keywords:** genetic counselors, education, workforce, disparities, Southern U.S.

## Abstract

The expansion of genetic and genomic testing across medical specialties and the changing workforce demographics of certified genetic counselors (CGCs) have led to concerns of a workforce shortage. We assessed the number of genetic counselors working in the Southern United States—a rural and medically underserved region—using various online and professional resources. We identified 683 practicing genetic counselors across the Southern U.S. and 160 specializing in prenatal genetics. CGCs were concentrated in urban areas; counties with a CGC had a significantly higher proportion of minority residents and median household income than counties without a CGC. There is an average of 2.97 prenatal CGCs per 5000 high-risk births in the South. Alternative delivery models are needed to increase access to counseling services in the Southern U.S., particularly for low income households and those of high risk pregnancies. Increased provider education and patient educational materials can help facilitate informed decision-making in prenatal settings as genetic technologies gain a stronger foothold and bring value to medical practice.

## 1. Introduction

Advances in genetics and genomics research, new testing technologies, electronic medical records, and general public interest have combined to usher in the precision medicine movement. Yet the expansion of genetic and genomic testing may pose greater burdens on providers, particularly on genetic counselors, to adequately educate patients and promote informed decision-making. In particular, the limited number of certified genetic counselors (CGCs) and the lack of diversity in the profession has raised concerns regarding access and widening health disparities in light of increasing demands [1,2]. CGCs are specially trained to educate patients about their options for testing, communicate test results, and provide support services.

It is estimated that more than 4000 genetic counselors are currently practicing in the United States [3,4]. Most CGCs practice in cancer (52%), followed by prenatal (41%) and pediatric (29%) settings, and are based in university medical centers (30%), public or private hospital or medical facilities (31%), and diagnostic laboratories (commercial, nonacademic) (18%) [5]. In 2008, 82% of counselors reported working in clinical settings and providing direct patient care [6], dropping to 59% in 2018 [5] and 2019 [7]. With a wider range of job opportunities in administration, research, public health, industry, and public policy [8], the number of counselors practicing in clinical settings is declining [7,9].

Although it has long been widely recognized that there are disparities in access to genetic counselors [10,11], there has been little formal assessment of the shortage of certified genetic counselors [12] and the patient populations affected. In 2014, Radford et al. briefly touched on the shortage of the genetic counselor workforce and its unequal distribution among rural and urban U.S. [13] in the 10 most populous states in the country. In 2017, Cohen et al. published a study of the CGC workforce in Indiana [14].

The Southern U.S. may experience greater problems with access to genetic counselors than other regions of the country. Approximately 123 million people, or 38 percent of the U.S. population, reside in the South [15]. Approximately 20% of adults in the South reported fair or poor health status in 2014, compared to 16% in the Midwest and Northeast and 17% in the West [16]. A ranking of health systems reported that 11 of 13 states in the bottom quartile were based in the South [17]. The lower health status may be due to the fact that nine out of the top 20 states with the highest number of medically underserved areas (MUAs) are located in the South [18].

In this study, we examined the availability of genetic counselors working in the Southern U.S. and the demographics in areas with and without practicing CGCs. We specifically explored the accessibility of prenatal CGCs in this region, given the already limited access to prenatal care. Understanding the distribution of CGCs in the south US, and the populations that are affected can inform development of alternative delivery strategies to improve access to genetic services and health equity in CGC-underserved regions.

## 2. Materials & Methods

Research Design: We conducted a geospatial analysis of genetic counselors’ work location with Southern states’ population, level of urbanization, median household income, racial demographics, and number of births. Based on the U.S. Census Bureau’s defined regions, the ‘South’ includes 17 states/regions (AR, AL, DE, DC, FL, GA, KY, LA, MD, MS, NC, OK, SC, TN, TX, VA, and WV) [19]. The study obtained an exemption approval from the Duke University Campus Institutional Review Board (#2017-0652).

Data Collection: To identify where genetic counselors work, we used three databases. The National Society of Genetic Counselors (NSGC) website has a “Find a Genetic Counselor” tool [20] that helps patients find a genetic counselor by state/province, institution/organization, first and last name, and types of specialization. Second, we used the American Board of Genetic Counselor’s (ABGC) website “Find a certified genetic counselor” tool [21]. Third, we used “Find a Provider” tool available through Healthgrades [22]—a healthcare company with information about physicians, hospitals, and healthcare providers. With the “Find a Provider” tool, patients can find genetic counselors near their location and according to their insurance, patient reviews, and the counselor’s availability, gender, age, and specialty. We collected all data in spring 2017 and updated the NSGC data in fall 2018. At the time of data collection, there were no counselors listed in these databases for the state of West Virginia and no prenatal counselors listed for the state of Louisiana and thus, we suspected that these data were incomplete given that one or more academic medical centers exist in these states. We therefore reviewed the directories of academic medical centers in these two states to confirm whether our initial results were accurate. We identified additional counselors through these sites and added them to our analysis.

While several types of health professionals may provide genetic counseling services, such as clinical social workers, physicians, and nurses, we included only board-certified genetic counselors in our analysis. The NSGC tool explicitly notes whether the counselors are board-certified are not. The Healthgrades tool, however, is less clear about board certification and therefore, we confirmed board certification through the ABGC’s database.

Several data points for each counselor were recorded in Excel (Microsoft) file, including state, specialty (e.g., cancer and prenatal), whether they offered telecounseling or see patients in-person, their employer, and their zip code. We did not store names or personal contact info nor did we contact any genetic counselors identified in the search. We recorded five-digit zip codes in a separate excel spreadsheet and matched each zip code with its respective county and latitude and longitude coordinates in order to import the data to GIS mapping tool.

Identifying State/County Demographics: To identify the demographic characteristics of counties in the southern U.S., we relied on data from the American Community Survey (ACS) of the U.S. Census Bureau [23]. We used the 5-year estimate (2012–2016) dataset of the ACS at the county geographic level rather than the 1-year dataset because the 5-year dataset has the largest sample size and is considered more reliable, particularly for small populations and analyzing census tracts. The Bureau defines Urbanized Areas as those having 50,000 or more people.

We recorded data on total state and county populations to calculate the number of CGCs per 1,000,000 residents in each state and the median population of counties with and without CGCs. To assess income, we used ACS county and state level data on “Median Household Income in the Past 12 Months (in 2016-Inflation-Adjusted Dollars).”

In addition, we used ACS data on race to examine the racial make-up of counties with and without practicing genetic counselors. The ACS classifies the racial demographics of a state and county into seven categories: “White alone”, “Black or African American alone”, “American Indian and Alaska Native alone”, “Asian alone”, “Native Hawaiian and Other Pacific Islander alone”, “Some other race alone”, and “Two or more races”. Given the low prevalence of some populations in the South we grouped “American Indian and Alaska Native alone”, “Asian alone”, “Native Hawaiian and Other Pacific Islander alone”, and “Some other race alone” into one category—“Some other race alone”. We also renamed “Two or more races” to “Mixed race” for clarity.

Finally, we used the ACS’s state-level data on "Women 15 to 50 Years Who Had a Birth in the Past 12 Months by Marital Status and Age". The data are divided into three age groups: “15 to 19 years old”, “20 to 34 years old”, and “35 to 50 years old”. We recorded the total number of births across all age groups and the total number of births specifically for women over 35 years old, as these would be considered high risk pregnancies and typically referred to genetic counseling according to clinical guidelines [24,25,26,27,28]. We created a pivot table to sum the number of women who gave birth in the past 12 months for ages 35 to 50 years old.

Data Analysis: Summary statistics were computed for population density, income, and race prevalence. We used ArcGIS Pro (ESRI) to create a regional map of CGCs’ practice location across the South after converting each CGC’s zip code location into geographic coordinates (latitude and longitude).

## 3. Results

**Figure 1:**
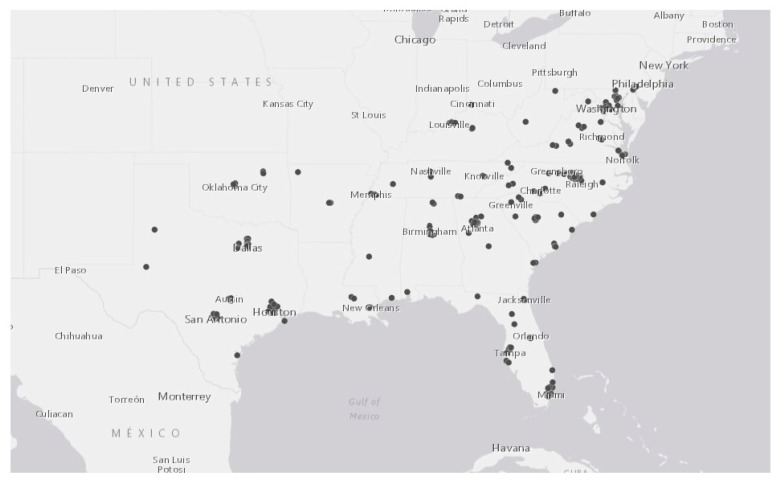
The distribution of certified genetic counselor’s workplace in the 17 states of the South.

Number of practicing genetic counselors: We identified a total of 683 practicing CGCs in the Southern United States (Figure 1). A total of 537 were identified through the NSGC site; we identified an additional 146 counselors through Healthgrades. According to the ABGC, there are 1015 board-certified CGCs across the 17 states. The 332 CGCs that we did not identify may be retired or no longer practicing, or may choose not to list their information on the ABGC. As shown in Table 1, the number of CGCs ranged from 2 to 115 (mean: 40; median: 20). Texas has the highest number of practicing CGCs with 115, and West Virginia the lowest with two.

Overall Access to Genetic Counselors by State & County: To assess workforce density and general access to CGCs, we first assessed state population data to determine the number of CGCs per 100,000 residents in each state (Table 1). The average number of CGCs per 100,000 residents in the Southern U.S. was 0.69. The three states with the largest populations in the South (Texas, Florida, and Georgia) had among the lowest number of practicing counselors per 100,000 people. Texas, having the largest population, ranked 10th out of the 17 states, with 0.43 counselors per 100,000 people. Florida—the second largest state by population in the South—ranked 14th out of the 17 states with 0.26 counselors per 100,000 people.

In contrast, the smallest populated states in the South had the highest number of practicing counselors per 100,000 people. Namely, the District of Columbia had 2.43 counselors and Delaware with 1.18 counselors per 100,000 people. One exception was West Virginia—the third smallest state in the South (by population) had the lowest number of counselors (0.11) per 100,000 people.

Geographical Proximity: We identified the county of practicing CGCs based on zip code of practice location and used this as a measure of access based on geographical proximity. However, we recognize that counties may differ with respect to size, public transportation, walkways, and development, which may impact accessibility to GCGs for residents. As shown in Table 1, CGCs were located in a total of 96 counties (out of 1383) and tend to be concentrated on average in five counties per state (range: 1–15), accounting for 15 percent (MS) to 100 percent (DC) of the population. The counties with practicing CGCs are densely populated, compared to counties without CGCs (only four out of 96 counties with CGCs have a population below 50,000, the U.S. Census Bureau’s cut-off for designation as a rural county, all located in Virginia) (*p* < 0.001).

Income & Race: We assessed the racial demographics and income of counties with and without practicing CGCs (Table 2) and found that counties with a CGC had a significantly larger proportion of minorities than counties without a CGC (*p* < 0.001). For example, in Georgia, Louisiana, and Mississippi, the proportion of minorities in counties with a CGC was greater than 50%, while the proportion of minorities in counties without a CGC was less than 35%. South Carolina was the only state where the proportion of minorities was larger in counties without a CGC (34%) than in counties with a CGC (31%).

With respect to income, we found that counties with CGCs had a significantly higher median household income than those counties without a practicing CGC (*p* = 0.00019) (Table 2). Median household income between counties with and without a CGC differed by a low of about $1500 (Virginia) to a high of about $20,000 (Georgia). In six of the 17 states in the South, median household income in counties with a practicing CGC was at least 20% higher than median household income in counties without a practicing CGC—Alabama (30.04%), Arkansas (22.13%), Georgia (34.69%), Kentucky (22.07%), South Carolina (22.06%), and Texas (23.25%).

Access to prenatal genetic counselors: It is difficult to estimate the appropriate number of CGCs given the wide range of practice settings and clinical indications for their services. Since state population data of the number of births from advanced maternal age pregnancies are available, we narrowed our analysis to practicing prenatal CGCs in each state in order to assess need versus supply. As counseling is indicated for other high risk pregnancies for a history of miscarriage or family health history, we will underestimate the high risk prenatal population warranting counseling services. Table 3 shows a comparison between the number of births (total) and births from advanced maternal age pregnancies in the last twelve months to the number of practicing prenatal CGCs in each state. There is an average of 2.97 prenatal CGCs per 5000 high-risk births in the South (range: 0.61–11.31). The districts of Columbia and Maryland have the highest percentage of high risk births (32.47% and 26.31%, respectively) and, similarly, had the highest number of prenatal CGCs per 5000 high-risk births. Florida and Georgia also had a high percentage of high risk births (23.11% and 20.32%, respectively), but a relatively lower number of prenatal CGCs per 5000 high risk births (1.20 and 1.58 prenatal counselors, respectively).

## 4. Discussion

As the field of genetics and genomic clinical applications continues to rapidly expand, access to expert providers is critical to the appropriate utilization of these applications, particularly CGCs [11,12,29]. The number of CGCs is considered below current needs [4] and our targeted analysis of southern states in the U.S. confirms the limited availability of CGCs in this region overall, and specifically, prenatal CGCs (Louisiana, Mississippi, and Arkansas). CGCs are concentrated in urban settings, and are accessible to a diverse group of patients residing in these areas with respect to race. However, CGC accessibility declines outside of urban centers, notably in rural counties and populations with low median household income.

The concentration of CGCs in urban areas is likely no different than other regions in the U.S., as this is typically where academic medical centers and large medical practices or hospital centers are located. Since CGCs cannot always bill for their services and receive reimbursement [29], they typically practice alongside physicians as part of a team to secure reimbursement for their services through the physician. Thus, changes in billing policies may result in better distribution of CGCs outside of academic medical centers into more community-based settings. However, ~28 million people reside in rural areas in the South and are of low income, and, thus, accessing CGCs that are hours away is not likely a feasible.

The southern states continues to face challenges regarding access to maternal care and poorer maternal and child outcomes. Access to prenatal care in the southern US is limited, particularly in rural areas, with the number of board-certified obstetrician/gynecologists decreasing [30,31] and a higher number of practicing nurse practitioners and family physicians providing care [32,33]. Many states in the southern US show high rates of pregnancy complications and death [34]. As the child-bearing age in the U.S. is increasing—with almost 10% of women having their first child at 35 years of age or older—the age at which women are considered to be high-risk [35], we were especially interested in exploring the accessibility of prenatal CGCs in the South. With about three CGCs per 5000 high risk births in the South and nine states with less than five prenatal CGCs, many women over 35 years of age will not have access to CGCs during their pregnancy. The use of noninvasive prenatal screening for chromosomal aneuploidies, initially for high-risk pregnancies, but increasingly offered to all expectant mothers [36,37,38,39], requires patient education to promote informed decision-making.

One potential approach to increasing access to genetic services, especially for patients living in rural areas, is telehealth or telemedicine [40,41,42]. Telemedicine is increasingly being incorporated in both primary care and tertiary care [43]. Genetic counselors have used telegenetics when in-person counseling is prohibitive due to time and travel costs [44,45] or to fill workforce shortages [46]. Counselors [47] and patients have reported satisfaction with telegenetics (or equivalent to in-person counseling) [42,45,48,49,50,51,52,53]. Areas in the south, such as Mississippi, have established telegenetics clinics across rural areas in the state to reduce barriers to care with comparable quality to in-person counseling sessions, with greater access and convenience [54]. Despite the broader outreach and convenience, telegenetics has some limitations including diminished rapport with patients, potential to miss patients’ emotional responses or clinical features [40,44,47], difficulties with billing and reimbursement [44], and CGC familiarity or comfort with phone or computer-assisted counseling (e.g., Skype) [55]. Other potential alternatives to address gaps in CGC access are group-based counseling sessions [56,57] or inclusion of genetic counseling assistants to increase patient volume [58].

As many health providers are struggling to keep up with the scientific advances and new clinical applications in precision medicine [59,60], educational tools will also be important to enable providers to understand and communicate with patients about new technologies [61]. Moreover, in the absence of a CGC, patient educational resources are essential to promote informed decision-making and communicate highly technical information, particularly in a time-sensitive setting with prenatal care [62,63,64]. However, patients may not be able to identify these resources on their own and therefore, will rely on their provider for guidance and direction [65]. Increased awareness of genetics by rural practitioners may also support use of rural genetic counseling clinics [66] and telegenetic services. Furthermore, partnerships between genetic and nongenetic providers can improve awareness and access to genetics care as needed [67,68] as well as institutional partnerships with groups working in underserved areas [69]. Reimbursement from insurers and Medicaid may allow non-CGCs to provide counseling services [70].

Interestingly, we identified (but did not include in the analysis) many health providers that were not board-certified, but that self-reported they could provide genomic services or genetic counseling. It is possible that these health professionals (mostly licensed clinical social workers, nurses, or Masters in Genetic Counseling but not board-certified) are filling in gaps where we observed shortages of CGCs. However, the differences in training and lack of certification may yield differences in quality and scope of counseling practices offered by non-CGCs.

The limited and unequal geographic distribution of CGCs across the South may impact access to prenatal screening and other needed counseling services. A crucial step to reducing the health disparities gap in genomics will be to improve access to expert providers, as well as educational resources for both nongenetics providers and patients. While developing and implementing alternative delivery strategies will prove crucial to closing the health disparities gap in the genomics field, some thought should also be given to the establishment of new genetic counseling training programs in the Southern US. Of the 45 genetic counseling training programs in the U.S. in good standing, 13 are based in the southern U.S. [71] and none of the three programs under development are located in the South. Increasing the number of training programs in the southern US may lead to an increased number of practicing CGCs in this region, as providers may be more likely to remain in the area (e.g., ~40% of physicians actively practice in the same state where they received their undergraduate medical education) [72]. The limited racial and ethnic diversity of CGCs (90% are White [7]) may be a significant factor for much of southern US with the highest percentage of African-Americans and two states with a high percentage of Hispanics (Texas and Florida) [73]. Establishing training programs based in the southern US may help increase the number of minority applicants. Concordant patient-CGC race may improve patient trust, understanding, and satisfaction [74]. A diverse CGC workforce is also essential to deliver cultural competence care and sensitivity with respect to decisions on family planning. Minority healthcare providers are more likely to care for a larger proportion of underserved patients [75].

Some limitations of this study should be noted. The data sources used to identify CGCs practicing in the southern U.S. each have limitations. The NSGC “Find a Genetic Counselor” tool only includes genetic counselors that are members of the society, which makes it possible our study excluded prenatal genetic counselors that are not members of the society. Moreover, for a small number of cases, five-digit zip codes were not all available for all counselors in the NSGC list and two states had more than 100 genetic counselors, but the tool only lists the first 100 counselors. In addition, some counselors may be a member of NSGC but are not practicing. Genetic counselors may choose not to include their information in the ABGC directory. With respect to Healthgrades, how often the website is updated and if the information is confirmed is unclear. Some academic institutions may not list mid-level healthcare providers in their directory, and therefore we may be underestimating the number of practicing CGCs. Other employment settings such as genetic testing laboratories and telegenetics companies were not queried. Finally, we only considered the high risk category of AMA given the availability of data for this variable. However, there are many other risk factors that are important to consider in determining counseling needs for prenatal care.

With the expanding use of genetic and genomics technologies across medicine, accessibility to CGCs is critical. With the changing workforce demographics of CGCs and unequal geographic distribution of the current workforce, access is quite limited, and even more so in underserved regions of the U.S. Thus, greater effort is needed to increase access to CGCs, potentially through telemedicine and greater training of providers about genetics, patient resources, and referrals.

## Figures and Tables

**Table 1:** The number of practicing certified genetic counselors (CGC) per state in the Southern United States. Source: Population Data—American Community Survey (2012–2016).

State	Number of Practicing CGCs	Number of CGCs per 100,000 People	Number of Counties with a CGC/total Number of Counties	Percentage of Population Residing in Counties with a CGC (%)
Alabama	19	0.39	4/67	33.66
Arkansas	11	0.37	2/75	20.61
Delaware	11	1.18	1/3	59.06
District of Columbia	16	2.43	1/1	100.00
Florida	52	0.26	11/67	58.32
Georgia	46	0.46	7/159	38.57
Kentucky	20	0.45	3/120	27.98
Louisiana	12	0.13	3/64	27.20
Maryland	101	1.70	4/23	50.89
Mississippi	5	0.17	2/82	14.83
North Carolina	105	1.06	11/100	43.93
Oklahoma	14	0.36	2/77	35.98
South Carolina	35	0.72	8/46	48.51
Tennessee	47	0.72	8/95	45.83
Texas	115	0.43	12/254	56.31
Virginia	72	0.87	15/95	39.46
West Virginia	2	0.11	2/55	15.84

**Table 2:** Median household income of the counties with certified genetic counselors (CGC) in each state of the Southern United States.

State	Median Household Income ($) of Counties without a CGC	Median Household Income ($) of Counties with a CGC	Percentage Difference in Median Household Income
Alabama	$37,557	$53,685	30.04
Arkansas	$36,032	$46,272	22.13
Delaware	$54,701	$66,283	17.47
Florida	$42,561	$49,196	13.49
Georgia	$38,436	$58,851	34.69
Kentucky	$39,479	$50,661	22.07
Louisiana	$40,771	$49,457	17.56
Maryland	$64,666	$80,454	19.62
Mississippi	$34,744	$40,934	15.12
North Carolina	$40,260	$51,232	21.42
Oklahoma	$44,216	$49,820	11.25
South Carolina	$37,362	$48,059	22.26
Tennessee	$38,854	$48,144	19.30
Texas	$45,557	$59,354	23.25
Virginia	$49,160	$50,727	3.09
West Virginia	$38,703	$46,794	17.29

**Table 3:** The total number of practicing certified genetic counselors (CGCs) that specialize in prenatal genetics and the number of births in the past 12 months (for all ages and for women aged 35–50, categorized as high-risk) for each state of the Southern United States. Note: Given that specializations were only available for data from the National Society of Genetic Counselors (NSGC), the table only reports figures for the counselors that were identified through the NSGC.

State	Number of Practicing Prenatal CGCs Listed in the NSGC	Percentage of High Risk Births (%)	Number of Prenatal CGCs per 5000 High Risk Births
Alabama	1	13.81	0.61
Arkansas	3	12.86	2.93
Delaware	1	23.57	1.90
District of Columbia	7	32.47	11.31
Florida	12	23.11	1.20
Georgia	9	20.32	1.58
Kentucky	3	15.71	1.71
Louisiana	2	14.06	1.17
Maryland	24	26.31	6.10
Mississippi	1	13.90	0.94
North Carolina	23	19.90	4.70
Oklahoma	3	14.43	1.98
South Carolina	12	17.40	5.81
Tennessee	4	15.40	1.59
Texas	35	18.90	2.33
Virginia	19	23.14	3.84
West Virginia	1	12.85	2.03
